# Mechanical stimulation in plants: molecular insights, morphological adaptations, and agricultural applications in monocots

**DOI:** 10.1186/s12915-025-02157-3

**Published:** 2025-02-25

**Authors:** Annalene Hansen, Agnieszka Gladala-Kostarz, Rebecca Hindhaugh, John H. Doonan, Maurice Bosch

**Affiliations:** 1 Institute of Biological Environmental and Rural Sciences (IBERS), Aberystwyth University, Aberystwyth, Gogerddan UK; 2 Department of Biosciences, Durham University, Durham, UK; 3 Newbury, UK

**Keywords:** Thigmomorphogenesis, Cereal crop mechanobiology, Grasses, Lodging resistance, Crop resilience to mechanical stress, Cell wall remodelling

## Abstract

Mechanical stimulation, including wind exposure, is a common environmental factor for plants and can significantly impact plant phenotype, development, and growth. Most responses to external mechanical stimulation are defined by the term thigmomorphogenesis. While these morphogenetic changes in growth and development may not be immediately apparent, their end-results can be substantial. Although mostly studied in dicotyledonous plants, recently monocot grasses, particularly cereal crops, have received more attention. This review summarizes current knowledge on mechanical stimulation in plants, particularly focusing on the molecular, physiological, and phenological responses in cereals, and explores practical applications to sustainably improve the resilience of agricultural crops.

## Mechanical stimulation: an underappreciated force affecting plant development and adaptation

Despite their sessility, plants possess an extraordinary ability to perceive and respond to their environment. Numerous environmental factors, such as light, temperature, water availability, and nutrient levels, profoundly influence plant growth and development. Among these, mechanical stimulation, an often underappreciated factor, can induce dramatic changes in plant phenotypes. For the purpose of this review, mechanical stimulation mainly refers to forces arising from external sources, predominantly wind and precipitation, but also including touch, gravity, and vibration/sound. The responses to such external stimuli are generally known as thigmomorphogenesis, which manifest in a range of morphological changes and physiological adjustments [1, 2]. Mechanical stimulation can also occur endogenously, mediated by the plant’s own growth, cellular movement, division, and morphogenesis [3]. Endogenous mechanical stimulation will not be explored in this review as there is a substantial amount of literature in this field (see e.g., [4–8]). Similarly, thigmotropism (directional growth or movement in response to touch), such as tendril coiling, and thigmonasty (rapid movements not dependent on the direction of the stimulus), such as the closure of a Venus flytrap (*Dionaea muscipula Ellis*), falls beyond the scope of this review. Instead, we focus on thigmomorphogenesis and the broader, long-term morphological and physiological adaptations to mechanical stimuli, with a particular emphasis on monocot grasses including cereals.

The ancient Greeks recognized that mechanical stimulation could affect the growth and development of plants. As early as 300 BC, the Greek philosopher Theophrastus observed that trees growing in windy environments were shorter and had denser wood compared to those growing in sheltered areas [9]. In this context, “wind” primarily refers to airflow rather than the effect of wind-carried particles, though such particles may add abrasive stresses in specific environments. During the late 20th and early twenty-first centuries, various types of mechanical stimulation have been imposed on plants to investigate the thigmomorphogenic response in both above- and below-ground tissue. These stimuli include water-spray to simulate precipitation, wind, brushing or rubbing to simulate touch, sound, vibration, gravity, and wounding (reviewed in [10]). Brushing and rubbing are commonly used as surrogates for natural stimuli like wind and typically involve calibrated, repeated physical contact to mimic the forces plants experience in their environments. Despite some responses being idiosyncratic to specific plant species [11, 12], common thigmomorphogenic phenotypes include reduced plant height, decreased shoot elongation and stem length, decreased above-ground biomass, increased uniformity, increased radial expansion, lignification of the stem, and increased stem and petiole flexibility [1, 11–19].

The discovery of touch-inducible genes (*TCH* genes) in 1990 [20] was seminal, offering an initial glimpse into the complex molecular underpinnings of thigmomorphogenesis. Subsequent research has elucidated some of the molecular signaling cascades and networks involved, particularly in Arabidopsis (*Arabidopsis thaliana*). This focus on dicots likely stems from the availability of well-established model organisms, such as Arabidopsis, which have been extensively studied due to their genetic tractability and experimental convenience. However, dicots and monocot grasses exhibit notable differences in some of their structural and compositional traits, which highlights the need for dedicated research into the responses of monocots to mechanical stimulation. Despite their importance in food security and ecology, these responses remain relatively underexplored.

**Fig. 1:**
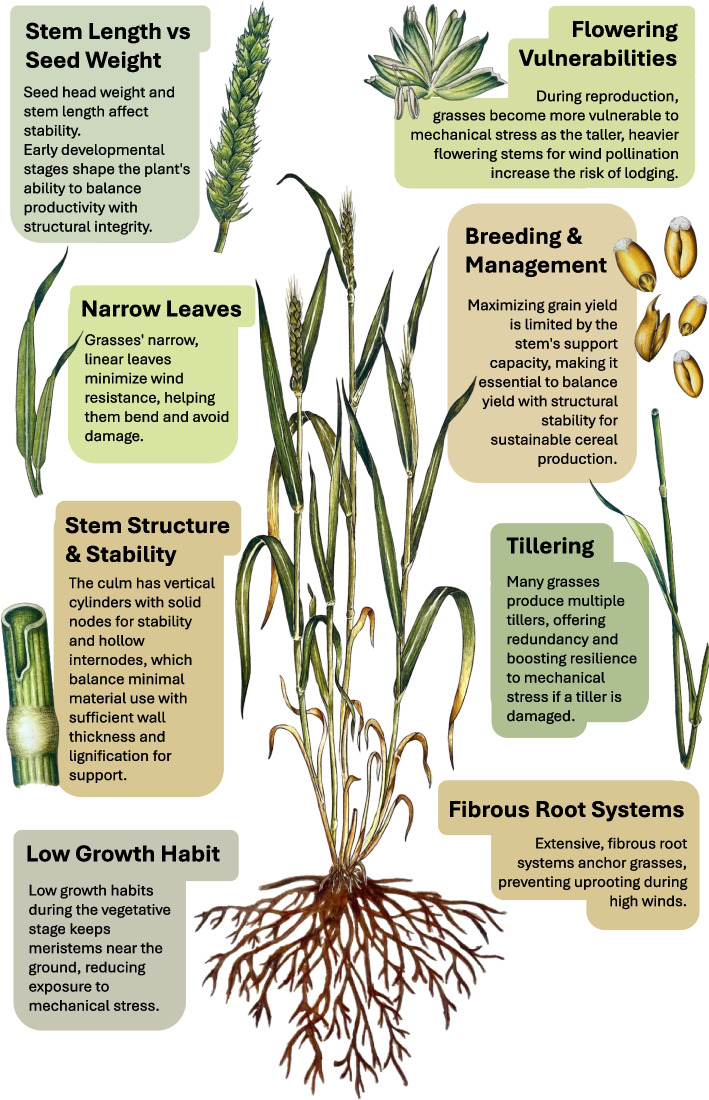
Anatomical and physiological adaptations of grasses influencing their responses to mechanical stimulation. Key adaptations of grasses that affect their response to mechanical forces are highlighted. Features such as stem structure, leaf shape, tillering, and root systems contribute to the resilience of grasses against mechanical stress, while vulnerabilities during the reproductive stage highlight the trade-offs between growth and stability. These adaptations balance productivity with structural integrity

As our climate changes, the prevalence and intensity of mechanical forces such as wind and rain are expected to increase [21]. This makes it essential to understand how plants, especially cereal crops, respond to these stimuli. Grasses are particularly well-adapted to withstand mechanical stresses, including wind, especially during their vegetative stages. Several anatomical and physiological traits contribute to both their resilience and potential vulnerability (Fig. 1).

In its most extreme form, mechanical forces can lead to lodging, where cereal crops are bent or flattened to the ground, resulting in dramatic yield losses. Increasing resistance to lodging is therefore a major trait for cereal crop improvement. While lodging can be regarded as a mechanical failure rather than an example of thigmomorphogenesis, it represents an extreme response to mechanical stress and is thus an important aspect to include in this review. We will provide a brief overview of lodging and genetic interventions and management strategies that can improve lodging resistance. While mechanical stimulation can have negative effects, it also holds significant promise for agriculture by promoting beneficial adaptations in plants. In vertical farming contexts, where crops are grown in stacked layers within controlled indoor environments to maximize space and resource efficiency, the dwarfing effects induced by mechanical stimulation are desirable. It has also recently been shown that repeated mechanical stimulation can increase antioxidant compounds and sensory profiles of leafy vegetables and basil, respectively [22, 23]. For cereals, the dwarfing and cell wall strengthening mechanisms induced by mechanical stimulation [24] may provide a tool to ameliorate lodging frequency and severity. The practical applications of mechanical stimulation in agriculture extend beyond preventing lodging. For instance, the enhanced structural integrity of plants following mechanical stimulation can make them more resistant to environmental stresses. The agricultural applications of mechanical stimulation are diverse and could even lessen the reliance on chemical inputs. We will discuss the potential of mechanical stimulation to prime or condition cereals to improve their resilience against environmental stresses.

## The molecular underpinnings of thigmomorphogenesis

**Fig. 2:**
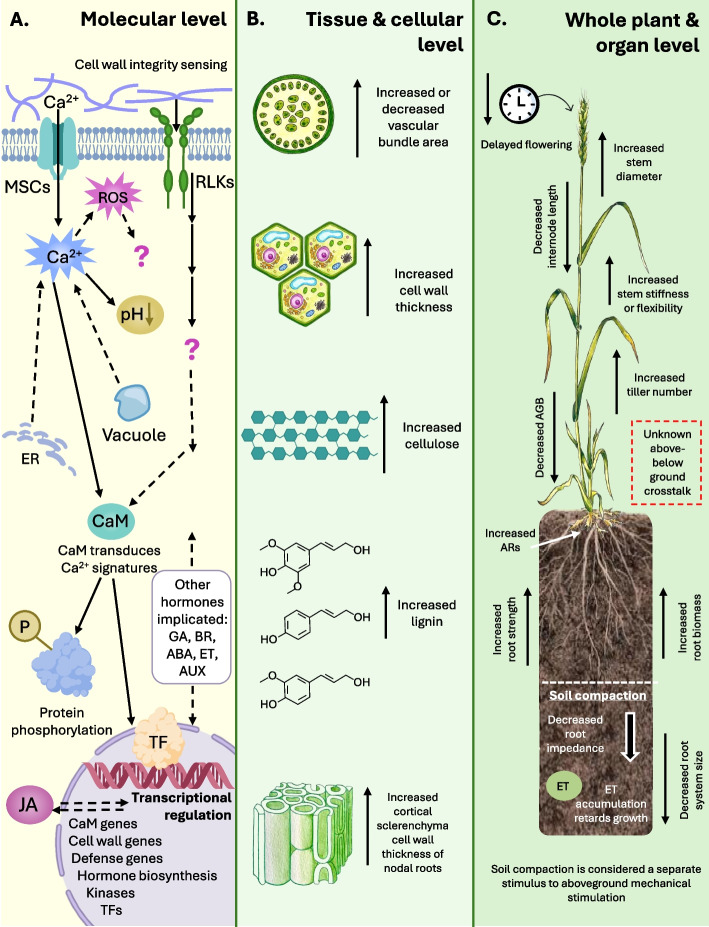
Responses of grasses to mechanical stimulation. Mechanical stimulation induces various phenotypic changes across plant, organ, tissue, and cellular levels. However, the molecular mechanisms driving these morphogenetic changes in grasses are not fully understood. The current model of mechanosensing and signal transduction, which leads to transcriptional and posttranslational modifications affecting thigmomorphogenesis, is primarily based on research from dicot model species. The figure illustrates the effects of mechanical stimulation across three levels. At the molecular level **A**, mechanical signals are perceived through mechanosensitive channels (MSCs) and receptor-like kinases (RLKs), triggering a cascade of intracellular events, including increases in calcium (Ca^2⁺^) and reactive oxygen species (ROS). Calmodulin (CaM) decodes the Ca^2+^ signatures, which along with the involvement of various hormones such as jasmonic acid (JA), gibberellins (GA), brassinosteroids (BR), ethylene (ET), abscisic acid (ABA), and auxins (AUX), leads to transcription factor (TF) mediated transcriptional changes that helps shape the thigmomorphogenetic response. At the tissue and cellular level **B**, mechanical stimulation can increase or decrease vascular bundle area, promote cell wall thickening, and enhance the synthesis of cell wall components like cellulose and lignin. Cortical sclerenchyma and cell walls of nodal roots also become more robust, enhancing the structural support. Mechanical stimulation impacts overall plant architecture **C**, affecting traits such as stem diameter, internode elongation, tiller number, and root architecture. Aboveground biomass (AGB) is typically reduced, while adventitious roots (ARs) increase, enhancing the overall resilience against mechanical forces. Interactions between aboveground and belowground responses, as well as the effects of soil compaction, further modulate the plant’s adaptive responses to mechanical stimulation

Mechanical stimulation triggers a cascade of responses. Among these, transcriptomic alterations represent a critical step, as they collectively drive the morphological and physiological changes characteristic of thigmomorphogenesis. Stress signals generated by mechanical stimulation are detected by a variety of mechanosensors, which convert these signals into complex downstream signaling pathways, initiating changes in gene expression, phytohormone signaling, protein phosphorylation, and metabolic feedback, ultimately resulting in a range of morphological adaptations [25, 26]. In this section, we will provide a brief overview of some of the key molecular components and events that have been identified in dicots (Fig. 2A), predominantly Arabidopsis. While many of these pathways are likely conserved across plant species, the precise molecular mechanisms involved in thigmomorphogenesis are still being elucidated. For a more detailed exploration of the molecular and cellular mechanisms underpinning this phenomenon, we refer readers to recent comprehensive reviews on the topic [25–28].

### Mechanoperception

Mechanoperception, the ability to sense and respond to mechanical stimuli, involves many components, including cell walls, mechano-sensitive ion channels (MSCs), receptor-like kinases (RLKs), and the cytoskeleton [27, 29–31]. One of the earliest detectable responses to mechanical stimulation is the generation of action potentials across the plasma membrane [19]. The deformation of the cell membrane triggered by mechanical stimulation activates force-sensing MSCs. These proteins, embedded in the cell membrane, act as gated conduits facilitating ion influx from the apoplast, particularly Ca^2+^, in response to mechanical stimulation, altering the membrane potential and ultimately allowing for signal transduction and cell-to-cell communication [32]. Various MSC families are implicated in mechanoperception including homologs of bacterial mechanosensitive channels of small conductance (MscS-like), Mid1-complementing activity proteins (MCA), two-pore potassium protein channels (K2P), reduced hyperosmolality-induced [Ca^2+^] increase (OSCA), and Piezo channels [25, 28, 29].

In addition to MSCs, RLKs play an important role in transducing mechanical signals into biochemical responses. RLKs have an extra-cellular domain that recognizes specific ligands including cell wall components, a membrane-spanning domain, and a cytoplasmic kinase domain responsible for initiating intracellular signaling cascades. This structural configuration positions RLKs as ideal candidates for mechanosensors, bridging the extracellular mechanical environment and intracellular biochemical machinery [33]. Among the various RLKs, members of the *Catharanthus roseus* RLK1-Like (CrRLK1L) family have been implicated in mechanoperception. FERONIA (FER) and THESEUS1 (THE1) are notable examples. FERONIA, in particular, has been shown to be crucial for Ca^2+^ signaling in response to mechanical stimuli such as touch and bending [34]. Arabidopsis mutants lacking FER exhibit impaired calcium signaling and reduced expression of touch-responsive genes, highlighting FER's essential role in mechanotransduction [34]. The extracellular domains of FER and other CrRLK1Ls, like THE1, display homology to animal malectin domains, which are involved in carbohydrate binding, suggesting their potential to bind cell wall polysaccharides. This binding ability supports the hypothesis that these RLKs can monitor cell wall integrity and mechanical changes, acting as cell wall sensors. In vitro studies have shown that FER can bind to pectin, a key cell wall component, which could enable it to detect and respond to cell wall deformations [35]. Besides being an activator of responses to mechanical stimulation, FER has also been shown to have a repressive role in the regulation of the jasmonic acid (JA)-dependent response to mechanical stimulation [29]. This may be because of differences in the type of mechanical stress imposed or the organ being studied. Other CrRLK1Ls [36] and members of other RLK families [37–39] participate in various processes like cell expansion, tip growth, and cell wall modification, which involves changes in cell biomechanics, and it is possible that some of these other RLKs are mechanosensory although this is yet to be demonstrated.

### Calcium and ROS signaling

Mechanical stimulation in plants triggers rapid increases in cytosolic Ca^2+^, which appear to be primarily facilitated by the influx of Ca^2+^ through the aforementioned MSCs. However, earlier work in *Nicotiana plumbaginifolia* implicated wind-induced increases in cytosolic Ca^2+^ to be of intracellular origin [40]. This suggests that initial Ca^2+^ influx through stretch-activated channels in the plasma membrane may trigger calcium-induced calcium release from intracellular organelles [14]. It is still unclear if Ca^2+^ signatures, encoding information of defined stimuli and which are crucial for determining the appropriate physiological response to different mechanical stimuli, are solely a result of Ca^2+^ influx through the plasma membrane or if there is also a significant contribution from intracellular Ca^2+^ release. Calcium-binding proteins, such as calmodulin and calmodulin-like proteins, play a pivotal role in transducing these Ca^2+^ signatures. The expression of genes encoding these proteins, such as *TCH1*, *TCH2*, and *TCH3*, is rapidly upregulated following mechanical stimulation, highlighting their importance in early signaling events [20]. Other Ca^2+^ sensors potentially involved in the decoding of the Ca^2+^ signatures include calcium-dependent protein kinases and calcineurin B-like proteins [41, 42]. These calcium-binding proteins can transduce Ca^2+^ signatures into phosphorylation events, changes in protein–protein interactions, or regulation of gene expression [42].

Mechanical stimulation has also been shown to induce the production of reactive oxygen species (ROS) in Arabidopsis and tomato (*Lycopersicon esculentum*), among other species [43, 44]. The role of ROS in thigmomorphogenesis is underscored by studies showing that plants with altered ROS scavenging systems exhibit modified responses to mechanical stimuli. For example, tomatoes overexpressing glutathione peroxidase, an enzyme involved in ROS scavenging, show a significantly reduced thigmomorphogenetic response, indicating the importance of ROS in this process [45]. It has been suggested that ROS may activate Ca^2+^-permeable cation channels [46], further amplifying the Ca^2+^ signal. Further research is needed to elucidate the precise mechanisms by which Ca^2+^ and ROS signals are integrated and propagated within the plant. Moreover, mechanical stimulation has been shown to lead to apoplastic alkalinization and cytoplasmic acidification in Arabidopsis roots [47]. While the former will alter the cell wall properties, the importance of cytosolic acidification in regulating plant cellular signaling networks is becoming increasingly appreciated [48, 49] and needs to be further investigated in the context of mechanical stimulation.

### Transcriptional and posttranscriptional regulation

Mechanical stimulation triggers extensive transcriptomic changes; for instance, over 2.5% of the Arabidopsis genome and 6% of the poplar genome were inducible and differentially expressed respectively, following mechanical stimulation [50, 51], highlighting the complexity and importance of mechanosensory responses. Transcripts commonly upregulated by mechanical stimulation include those encoding various calcium-binding proteins, such as calmodulin and calmodulin-like proteins (e.g., *TCH1*, *TCH2*, and *TCH3*). Another prominent class of induced transcripts are those related to cell wall modification, including xyloglucan endotransglucosylase/hydrolases (XTHs) like *TCH4*, pectin esterases, and cellulose synthases. These enzymes are crucial for modifying cell wall architecture, thereby enabling plants to adapt their structural properties in response to mechanical stress. The upregulation of genes involved in disease resistance and defense responses is also notable, suggesting an overlap of common pathways activated by mechanical stimulation and other environmental stresses, such as pathogen attack or abiotic stress. Such crosstalk between different signaling pathways enables plants to mount a coordinated response to a variety of challenges, enhancing their overall resilience. Moreover, mechanical stimuli affect the expression of numerous kinase and transcription factor genes; over 10% of the genes encoding kinases and transcription factors were increased in touch-stimulated Arabidopsis plants [50, 52], indicating that signal transduction pathways and transcriptional regulation are heavily influenced by such perturbations [14, 50].

There is considerable variability in the temporal expression profiles, with changes in differential expression depending on the time after mechanical stimulation (see e.g., [50, 51]). Additionally, significant differences are observed across plant tissues and developmental stages [53]. Transcriptional responses also depend on the type and frequency of mechanical stimulation, highlighting the complex nature of these responses. Besides changes in expression profiles, it has been shown that proteome-wide protein phosphorylation is a rapid and broad response to mechanical stimulation in Arabidopsis and may play a critical role in the mechano-response pathways of plants [54].

### Hormonal response to mechanical stimulation

Plant hormones play an essential role in every aspect of plant life, including responses to mechanical stimulation. Most plant hormones, including JA, ethylene, abscisic acid (ABA), auxin, brassinosteroids, and gibberellins (GA), have been shown to be involved in thigmomorphogenesis [27]. These hormones can interact both antagonistically and synergistically to coordinate stress responses, maintaining homeostasis during thigmomorphogenesis to support growth and development [25].

JA is an integral component of thigmomorphogenesis. For instance, it was shown that mechanical stimulation dramatically increased the levels of a metabolic intermediate of JA biosynthesis in *Bryonia dioica* and bean (*Phaseolus vulgaris*) [55]. Likewise, mechanically stimulated Arabidopsis plants displayed a two-fold increase in JA levels. Moreover, loss of function mutants of allene oxide synthase, a key enzyme in JA biosynthesis, did not show typical thigmomorphogenic responses [56]. Mechanically induced JA accumulation is directly controlled by MYC2/3/4 transcription factors, through a positive feedback loop that regulates JA-biosynthesis and also triggers a network of downstream transcription factors and effectors involved in defense responses [57]. A Ca^2+^-induced signaling network is responsible for the systemic increases in JA levels induced by wounding. The de-coupling of an inhibitory complex of JA-synthesis genes is initiated by the perception of elevated Ca^2+^ levels by calmodulin. In a calmodulin-dependent fashion, phosphorylation of Jasmonate-Associated VQ-Motif Gene1 (JAV1) occurs, resulting in the disintegration of the inhibitory complex JAV1-JAZ8-WRKY51. This alleviates repression of allene oxide synthase transcription, leading to JA production [58, 59]. As there is a significant overlap in the transcriptomic responses between wounding and mechanical stimulation, a similar signaling network may be responsible for the increases in JA levels induced by mechanical stimulation. However, classic touch-inducible marker genes such as calmodulin-like *TCH2* and *TCH3*, and *TCH4*, an XTH family member involved in cell-wall modification, remain inducible by mechanical stimulation in Arabidopsis mutants defective in JA synthesis and signaling (e.g., *allene oxide synthase* and *myc2 myc3 myc4*) [56, 57]. This indicates that mechanical stimulation also triggers a JA-independent signaling pathway. Indeed, it has been found that calmodulin-binding transcriptional activators CAMTA1/2/3 activate the expression of JA-independent touch-responsive genes including *TCH2*, *TCH3*, and *TCH4* [29].

Levels of the phytohormone gibberellin are generally lower following mechanical stimulation, suggesting that GA depletion may be responsible for the generally observed growth inhibition induced by mechanical stimulation [11]. For instance, mechanical stimulation-induced growth retardation was restored by exogenous application of GA in cucumbers *(Cucumis sativus)* [60]. Similarly, in Arabidopsis, mechanical stimulation decreased gibberellin levels, but the induced morphological changes could be reversed through the application of a bioactive form of gibberellin [61]. Moreover, mechanical stimulation induced the expression of *AtGA2ox7*, which encodes a GA catabolism enzyme, potentially leading to decreased levels of GA. This is supported by loss-of-function mutants for *GA2ox7* not responding to mechanical stimulation [61]. As there is crosstalk between the phytohormone signaling pathways of JA and GA, a model was proposed on the balance between GA catabolism and JA accumulation that governs thigmomorphogenesis, with factors affecting this balance including duration and intensity of mechanical stimulation as well as the developmental stage of the plant [25].

A role for ethylene has been proposed based on the observation that mechanical stimulation of plants led to ethylene evolution and exogenous ethylene application resulted in thigmomorphogenic-like changes [62]. Ethylene levels increased in response to mechanical stimulation in both bean and pea (*Pisum sativum*) [63, 64]. Moreover, mechanical stimulation of mung bean (*Vigna radiata*) led to increased expression of 1-aminocyclopropane-1-carboxylic acid (*ACC*) while the expression of both *ACC* and 1-aminocyclopropane-1-carboxylate synthase (*ACS6*), along with key regulatory enzymes of ethylene biosynthesis, was increased in Arabidopsis [65, 66]. Nevertheless, ethylene involvement was questioned as Arabidopsis ethylene receptor mutants and tobacco (*Nicotiana tabacum*) ethylene-insensitive transgenic lines showed no significant morphogenetic changes in response to mechanical stimulation when compared to wild-type plants [62, 67]. Recent studies in Arabidopsis, however, have shown that ethylene is directly involved in mediating thigmomorphogenesis by regulating pectin degradation [68] and by modulating GA levels independently and antagonistically from JA [69].

Although data suggest the involvement of auxin, ABA, and brassinosteroids in thigmomorphogenesis, the extent of their roles remains to be fully elucidated. Studies have shown changes in the expression of ABA biosynthetic genes and reduced ABA levels following mechanical stimulation [57], as well as classic thigmomorphogenic phenotypes induced by exogenous ABA application [15]. Similarly, the expression of *TCH4*, encoding an XTH, is regulated by auxin and brassinosteroids [70], indicating their potential role in mechanical stress responses. Additionally, differential regulation of auxin-responsive genes has been observed following mechanical stimuli, although direct applications of auxin did not significantly affect thigmomorphogenesis in certain plant species [25]. Despite these findings, more research is needed to comprehensively understand the specific contributions of these hormones to thigmomorphogenesis.

## Mechanical stimulation in cereals: the knowns and unknowns

The grass family (Poaceae) includes all the major cereal crops such as wheat (*Triticum aestivum*), maize (*Zea mays*), barley (*Hordeum vulgare*), oat (*Avena sativa*), and rice (*Oryza sativa*). As these provide over half of the daily global calorific intake for humans, and also play an essential role in livestock nutrition, cereal crops arguably represent the most important plant species for human civilization [71]. The consequences of mechanical stimulation on the grasses, and cereal crops in particular, are therefore relevant to food security, a critical issue due to the continually growing global population and anticipated climate changes. Members of the grass family are among the most resilient to various mechanical stresses, especially when compared to many dicotyledonous species. Grasses often dominate wind-prone habitats, such as coastal and mountainous regions, and some species even exhibit tolerance to direct physical damage, such as animal trampling (e.g., Kentucky bluegrass (*Poa pratensis* L.) and perennial ryegrass (*Lolium perenne* L.)) [72, 73]. However, during reproduction, grasses are particularly sensitive to mechanical stimulation which has economic and food security implications. The heavy seed heads formed at the stem’s extremities present a significant physical challenge to plant stability (Fig. 1), affecting harvestability and quality traits. Despite these known factors, knowledge about the morphogenetic response of the grasses to mechanical stimulation and its impact on relevant agronomic traits remains limited, with a greater understanding and appreciation of its importance only recently emerging [16, 17, 74].

Similarly to dicots, the most widely observed responses of cereal crops to mechanical stimulation include decreases in shoot elongation, leading to shorter stems, and a general reduction in aboveground biomass (Fig. 2C). These effects have been observed in several cereals, including wheat, rice, maize, sorghum (*Sorghum bicolor*), as well as the model grass *Brachypodium* (*Brachypodium distachyon*) [16, 17, 75–77]. A reduction in stem length is strongly correlated with a decrease in internode length (Fig. 2C). For example, rubbing rice stems caused a decrease in the length of the second internode, though no differences were observed in the third and fourth internode [78]. In *Brachypodium*, mechanical stimulation reduced the length of most internodes [16], whereas in wheat and sorghum, reductions of particular internode lengths related to the age of the plant when mechanical stimulation commenced [17, 77].

In contrast to the more universally observed stem shortening, changes in stem diameter are more variable. For instance, increases in stem diameter were observed in sorghum after bending [77] and in rice after rubbing [78] while no changes were observed after mechanical stimulation in maize and *Brachypodium* [16, 79]. A recent study on wheat plants suggested that increases in stem diameter induced by mechanical stimulation may be age-dependent. An increase in stem diameter was observed in young wheat seedlings after brushing while no effect was observed after brushing of older seedlings [17].

While some studies have examined the effect of mechanical stimulation on the phenotypic traits of cereals, little attention has been given to how such stimulation may affect anatomical features, particularly those of stem tissues (Fig. 2B). Earlier work suggested that mechanical stimulation may be positively correlated with the number, area, and layout of vascular bundles in oats [80] and tall fescue (*Festuca arundinace*) [81]. More recent studies indicate that changes in anatomical features in response to mechanical stimulation may vary between genotypes within a species. For instance, the rice genotype Shengbasimiao showed no significant difference in vasculature after rubbing [78] while the genotype Simiaoxuan developed larger areas of vascular bundles [82]. A comparison of two *Brachypodium* genotypes also revealed differences in vasculature responses; mechanical stimulation decreased the area of both inner and outer vascular bundles in Bd21, while in ABR6 it increased the area of vascular bundles [16]. These findings indicate that morphological responses to mechanical stimulation may vary based on how and when the stimulation is applied and that there is genetic variation in responses not only between different species but also among genotypes within the same species.

Plant cell walls are highly dynamic and complex cellular structures supporting plant growth, development, physiology, and adaptation. They are primarily composed of the polysaccharides cellulose, hemicellulose, pectin, and the phenolic polymer lignin, but the abundance and organization of the different cell wall components differ depending on developmental stage, organ type, and cell type [83]. The sophisticated composite structures of plant cell walls are crucial for maintaining structural integrity and enabling plants to adapt to various stresses and environmental conditions. However, until recently, surprisingly little was known about how cell wall components change in response to mechanical stimulation. Mechanical stimulation of *Brachypodium* increased the cell wall lignin content by up to 40% when compared to controls. The abundance of several cell wall monosaccharides was also affected; particularly an increase in glucose, primarily derived from cellulose, and structural changes to pectins were observed. These alterations in cell wall characteristics induced by mechanical stimulation, which also included an increase in cell wall thickness (Fig. 2B), increased the resistance of the biomass to enzymatic sugar release [16], an important factor when considering suitability for biorefining into biofuels and commodity chemicals. The increased lignin content may increase the energy content of the biomass [84]. These aspects are relevant as the straw of cereal crops, and the biomass of dedicated biomass grasses such as *Miscanthus* and switchgrass (*Panicum virgatum*), can be used for renewable energy production and biorefining [85].

Anatomical features, stem diameter, and perhaps most importantly, cell wall characteristics, will affect the biomechanical properties of the stem. While studies on biomechanical properties in cereals have primarily focused on identifying lodging-resistant and lodging-susceptible variants [86–89], limited attention has been given to changes induced by mechanical stimulation. Nevertheless, mechanical stimulation increased the stiffness of stem internodes in *Brachypodium*, suggesting that changes in cell wall characteristics, in particular increases in lignin content, are associated with increased stiffness [16]. Additionally, studies on wheat and sorghum have shown varying responses to mechanical stimulation; wheat exhibited increased stem stiffness [17] while sorghum developed stems that were less stiff and more flexible [77]. The increased flexibility increased the force required to cause failure under bending tests, indicating that plants may resist greater external loads. This suggests that even within the Poaceae family, species have evolved different biomechanical adaptations to mechanical stimulation.

While most studies on the response of cereal crops to mechanical stimulation focus on plant growth and development, biomechanical aspects, and molecular signaling events, there is a notable lack of data on reproductive traits in contexts other than lodging. Grain production is a critical trait in cereal crops because of their economic value and one might expect a trade-off where more resources are allocated toward strengthening stems at the expense of reproductive output. Indeed, studies have shown that mechanical stimulation of young *Brachypodium* and wheat plants delayed flowering and reduced seed yield [16, 17]. However, more research is needed to fully understand the effects of mechanical stimulation on reproductive traits in cereals, including its impact on grain yield and quality.

Above-ground mechanical stimulation can also affect the below-ground root system, indicating long-range signal transduction. Roots of flexed maize plants were thicker and more numerous than those of plants that received no mechanical stimulation [79]. Moreover, besides the morphology of roots, their mechanical properties were also affected; roots were stronger, more rigid, and stiffer than those of unstimulated plants [79, 90]. Mechanical stimulation of perennial ryegrass increased the root biomass in conditions of low water availability [91]. More recently, windy conditions have been shown to induce the development of shoot-born adventitious roots (ARs; Fig. 2C), roots formed from non-root tissues, from the leaf nodes in *Brachypodium* [92]. These ARs make wind-acclimated plants less susceptible to lodging. The formation of ARs is mediated by auxin and is triggered by direct physical contact of the leaf nodes with soil particles. This initiates the transcriptional induction of the auxin-responsive transcription factors *WUSCHEL-related homeobox* (*WOX*) and *lateral organ boundary structural domain* (*LBD*) [92]. Another study showed that mechanical forces transduced from the shoot to the root stimulate cell wall thickening of the cortical sclerenchyma of nodal roots in *Brachypodium*, altering the root mechanical properties and improving resistance to lodging [93]. The effect of above-ground mechanical stimulation on below-ground roots, including root growth and architecture, requires further investigation to better understand these interactions and their implications for plant development and resilience.

In addition to the influence of above-ground mechanical stimuli, roots are also directly subjected to mechanical forces as they penetrate the soil, which significantly impacts their growth and development. Compacted soil, for example, could be considered a form of mechanical stimulation [26]. Decreased macroporosity in compacted soil leads to increased mechanical impedance and decreased fluid transport rates, resulting in reduced root growth and crop productivity [94, 95]. It should be noted that soil compaction not only physically limits root penetration, it also increases ethylene buildup in the rhizosphere, which further inhibits root growth. Interestingly, roots lacking the ethylene response pathway show improved growth through compact soil, highlighting potential avenues for crop improvement in increasingly degraded soils [96]. Soil compactness causes a reduction in root system size (Fig. 2C), often accompanied by thickening of the roots, anatomical changes, and root architecture alterations; this has been observed in cereals including wheat, oats, barley, and rice [97–101].

Most of our knowledge about molecular responses to mechanical stimulation has been derived from dicots, particularly Arabidopsis. While Arabidopsis is a valuable model for dicots, there are distinct differences between dicots and monocots related to their morphological and anatomical features as well as cell wall composition [102]. Hence, not all findings from dicots can be directly translated to monocot cereal species and our understanding of the molecular responses of cereals to mechanical stimulation remains very limited (Fig. 2A).

Early studies in the 1990s suggested that JA-dependent signaling occurs in cereals following mechanical stimulation, with increased JA levels observed after wounding in oats and an increase in the expression of lipoxygenase *LOX1*, involved in JA biosynthesis, after mechanical stimulation in wheat [103, 104]. Recent in-depth analysis showed that cereals are highly responsive to mechanical stimulation at the transcriptional level, with many key aspects of signaling and plant growth being affected [74]. The most prominent changes were detected 10–25 min after mechanical stimulation, with 1–2% of the transcriptome responding. The involvement of JA and other hormones such as ethylene, cytokinin, and auxin was observed in response to mechanical stimulation. Increased expression profiles of genes encoding enzymes involved in cellulose, hemicellulose, and pectin biosynthesis suggested that the composition of cell walls may be altered by mechanical stimulation in cereals. In contrast, genes related to lignin and phenylpropanoid biosynthesis were rapidly downregulated after mechanical stimulation in barley, oats, and wheat [74]. Additionally, several gene-families involved in cell wall modification not previously associated with responses to mechanical stimulation were upregulated in *Brachypodium*, including the glycosyl hydrolase (GH) family 17, involved in modifying β−1,3-glucans, GH18, encoding chitinases, and cellulose synthase-like F6 (*CslF6*), involved in (1,3;1,4)-β-glucan biosynthesis (mixed-linkage glucan) [105]. Mechanical stimulation of sorghum stems revealed enrichment of genes associated with cell wall biology, hormone signaling, and general stress responses in response to mechanical stimulation [24].

Another class of genes most affected by mechanical stimulation in cereals included orthologs of well-known touch-inducible genes from Arabidopsis such as the calmodulin-like *TCH2* and the XTH encoding gene *TCH4*, which were upregulated immediately following stimulation of oats, barley, and wheat [74]. Mechanical stimulation of *Brachypodium* roots also resulted in the upregulation of classic touch-responsive genes, including orthologs of the calmodulin (CaM) and calmodulin-like (CML) genes *TCH1-3* and the XTH *TCH4* [105]. While this indicates conservation of mechanical stimulation responsive gene expression between monocots and dicots, several cereal-specific genes induced by mechanical stimulation were identified. These include genes involved in suberin synthesis, a callose synthase, and several malectin-like domain receptor-like kinases [74]. Their results indicate that, similar to Arabidopsis, JA-dependent and JA-independent signaling pathways are activated in response to mechanical stimulation in cereals. A model was proposed in which Ca^2+^ or ROS are involved in the systemic spreading of touch-induced signals to other parts of the plant, resulting in various morphogenetic and defense-related responses [74].

In conclusion, significant progress has been made recently in identifying molecular components involved in the responses of cereals to mechanical stimulation, pointing towards mostly conserved but also unique signaling pathways compared to dicots. However, key questions remain, such as how these molecular responses translate into the observed morphological and structural adaptations triggered by mechanical stimulation and how these molecular pathways integrate with other environmental stress responses. Further research is needed to fully understand these processes and their implications for crop resilience and productivity.

## Mechanical stimulation and lodging

In its most extreme form, mechanical stimulation can cause mechanical failure and result in lodging, a permanent displacement of plant stems from their upright position, resulting in plants leaning horizontally [106]. This phenomenon is primarily caused by high wind-loading on above-ground plant tissues, exacerbated by rainfall or high moisture levels, but is also affected by topography, soil type, pests, disease, and husbandry practices [107, 108]. Cereal lodging mostly manifests in two primary forms: stem lodging, where the stem either bends or breaks, and root lodging, which results from the failure of the root system to maintain anchorage. The implications of lodging can be severe and include reductions in grain yield and quality, as well as difficulty with harvesting the crop, leading to significant economic losses. Studies highlight substantial yield reductions across various cereal crops due to lodging; for instance, wheat can suffer average yield losses of up to 25% annually in the UK, but in extreme lodging years 60–80% of yield may be lost [107, 109]. Similarly, global annual yield losses due to lodging in maize are estimated at 5–20% [110], in barley as high as 28–65% for the UK [111], and in rice up to 50% for Japan following heavy storms [112]. Despite these figures, quantitative data on the extent and impact of lodging in cereals is scarce, and the information available is geographically limited. More comprehensive data is needed to fully understand the broader impact of lodging on cereals. Questions remain about the prevalence of lodging, its exacerbation by climate change, with stronger winds and heavier precipitation being predicted [113], and the potential for improved prediction models. Advances in technology, such as unmanned aerial systems or satellite imagery combined with artificial intelligence and machine learning can offer promising methods to obtain more accurate data on the prevalence and impact of lodging [114, 115].

**Fig. 3:**
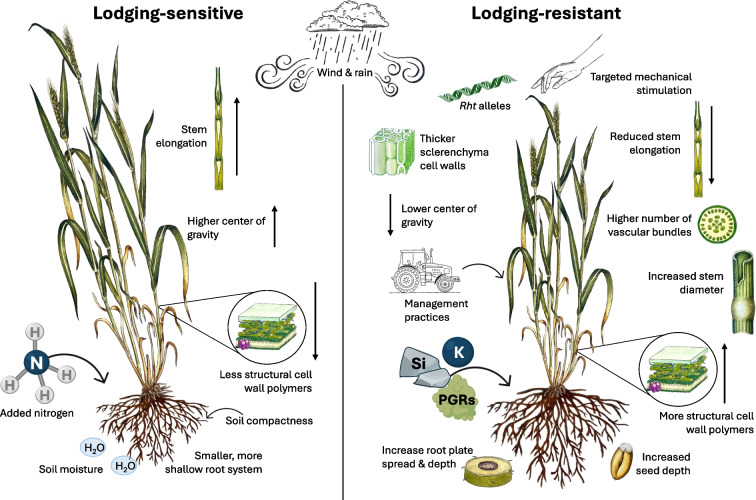
Factors contributing to lodging sensitivity and resistance in cereal crops. Key factors that influence lodging sensitivity and resistance in cereal crops are contrasted. Environmental conditions, nutrient availability, management practices, and structural characteristics such as cell wall composition and root systems are highlighted, illustrating how they collectively affect crop stability and resilience under mechanical stress. PGR, plant growth regulators; *Rht*, reduced height

Given the agronomic importance of cereals, significant progress has been made in developing varieties that reduce the incidence of lodging. Most research has focused on improving lodging resistance in wheat and rice, whereas other small grain cereals have received somewhat less attention. Plant height is a crucial factor determining susceptibility to lodging [116]. The final height of cereals is mainly a result of internode elongation, which is regulated by genes involved in the biosynthesis of gibberellins, brassinosteroids, and related signaling networks [117]. During the Green Revolution of the 1960s and 1970s, semi-dwarf wheat cultivars were developed with *Rht* (reduced height) alleles, which cause an insensitivity to GA. This resulted in shorter plants with an increased harvest index (ratio of grain to total shoot dry weight) and reduced lodging (Fig. 3). Dwarf and semi-dwarf varieties of oats, barley, and rice with increased lodging resistance have also been developed [107, 118]. Niu et al. provide an excellent summary of genetic improvements to reduce plant height, including dwarfing genes and their functions in cereals [117, 119]. Plant height is directly correlated with the center of gravity, with shorter plants having a lower center of gravity, which enhances stability and further reduces lodging risk. Despite advances in breeding modern dwarf and semi-dwarf cultivars that exhibit reduced lodging, lodging remains a prevalent challenge. Increasing lodging resistance, therefore, remains a major trait for cereal crop improvement. It has been suggested that the minimum height for optimal grain yield is being approached for modern cereal varieties [107]. Further height reductions may compromise photosynthesis and limit metabolic processes, potentially leading to yield stagnation or even reduction [116, 120]. Therefore, other plant traits may need to be targeted to increase lodging resistance without negatively impacting yield.

Stems need to be both strong and flexible to resist lodging. The material properties of the stem, which determine its mechanical strength and elasticity, are primarily determined by the cell wall composition and architecture, as well as the anatomical features of the stem. Lignin, a complex phenolic polymer tightly cross-linked with other cell wall components, plays a vital structural role in secondary cell walls. Its accumulation contributes significantly to the plant’s overall strength and increased lignin accumulation is typically associated with improved lodging resistance [121–124]. For instance, transcript profiling and metabolite analyses of stems from maize plants with different lodging susceptibilities identified several lignin biosynthesis genes and lignin-associated metabolites implicated in regulating lodging resistance [125]. Crystalline cellulose microfibrils are another important determinant of the mechanical properties of plant cell walls, with higher levels of cellulose crystallinity correlating positively with the breaking force of wheat stems [126]. It was recently shown that the increased expression of the transcription factor *OsTCP19* in rice, which increased cellulose biosynthesis and decreased lignin biosynthesis, improved lodging resistance without affecting grain yield [127]. However, it has also been reported that a reduction of cellulose crystallinity in rice caused by a mutation of a cellulose synthase gene (*CESA9*) improves lodging resistance [128]. Overall, it seems that while individual cell wall components can impact lodging resistance, it is the composite structure of the cell wall, primarily comprised of cellulose, hemicellulose, and lignin, and their respective interactions, that is the main determinant of the cell wall’s structural properties, integrity, and impact on lodging resistance (Fig. 3).

Anatomical and structural features of the stem also play a crucial role in determining lodging resistance in cereal crops [116, 126]. Specifically, a higher number of vascular bundles and increased cell wall thickening of sclerenchyma cells, particularly those located under the epidermis and crucial for bending stress resistance, appear to be important for increasing stem lodging resistance [129] (Fig. 3).

While stem lodging typically receives the most attention, root lodging is also a critical but often overlooked aspect of crop stability. For wheat, it has been suggested that varieties with greater root plate depth and a wider spread would improve anchorage, and thus exhibit reduced root lodging [130, 131]. Indeed, root plate spread from field-grown wheat plants correlated with low lodging incidence and was found to be highly heritable [132]. Additionally, traits such as higher root density in subsoil may enhance root lodging resistance. For example, in rice, tolerant varieties developed a larger amount of roots in deeper soil layers than susceptible varieties [133] (Fig. 3). However, the genetic basis of root lodging-related traits in cereals remains poorly understood.

The extent and impact of lodging can be minimized through effective crop management [107]. Agronomic practices such as reducing plant density, increasing seed depth, delaying the date of sowing, reducing tillage, reducing irrigation, and mixed crop cultivation have been found to reduce the incidence of lodging [130]. Crop nutrition, particularly the application of nitrogen fertilizer, plays a significant role in lodging susceptibility (Fig. 3). While high nitrogen application rates increase grain yield, they also increase the risk of lodging in cereals by weakening stem strength and reducing root anchorage [134]. On a molecular level, it has been shown that high nitrogen application rates down-regulate the expression of genes involved in lignin and cellulose biosynthesis, decreasing their deposition in secondary cell walls, particularly for lignin in sclerenchyma cells, thereby decreasing the mechanical strength and lodging resistance [121, 135, 136]. In contrast, the application of silicon, though considered a non-essential macronutrient for plants, has been shown to increase lodging resistance in both wheat and rice by improving stem strength [137, 138] (Fig. 3). Similarly, the application of potassium has been shown to increase the strength of rice and maize stems, significantly decreasing lodging [139, 140]. Thus, carefully considered fertilization measures can substantially mitigate some of the lodging risks in cereals.

Plant growth regulators (PGRs) are widely used in cereal crops to further reduce plant height and increase resistance to lodging [107, 124]. Two common groups of PGRs are inhibitors of GA biosynthesis and ethylene-releasing compounds, both of which reduce elongation and decrease the rate of cell division, resulting in shorter plants that are more resistant to lodging (Fig. 3). For example, the gibberellin biosynthesis inhibitor chlormequat is applied to most cereal crops grown in the UK for lodging control [141]. Interestingly, another GA biosynthesis inhibitor, paclobutrazol, not only increased lodging resistance by reducing plant height but also increased the accumulation of lignin in wheat and maize [123, 142]. However, while PGRs are effective in reducing the occurrence of lodging, their use raises environmental concerns and future regulations may limit their application [141]. Therefore, balancing the benefits of PGRs for lodging resistance with their potentially negative environmental impact is crucial for sustainable cereal production.

Mechanical stimulation imposed on young plants has been demonstrated to modify morphological, anatomical, and chemical properties that relate to lodging. Mechanical stimulation produces plants with reduced height, shorter internodes, and stronger stems with altered mechanical properties [1, 14, 24, 77]. Increases in lignin content in wheat have also been identified in response to mechanical treatment [143]. This therefore raises the question of whether mechanical treatment could be utilized as a form of thigmo-priming to improve crop resistance to lodging.

## Mechanical conditioning and agricultural applications

It is clear that mechanical stimulation triggers intricate signaling events that can eventually lead to a range of morphological adaptations in plants (Fig. 2). In this section, we explore potential agricultural applications of mechanical stimulation to improve the resilience of crops and thus enhance agricultural productivity and sustainability. The concept of using mechanical stimulation to enhance quality-related characteristics is well-documented in the horticultural sector. Various methods of mechanical stimulation have been employed to produce shorter, more uniform-looking plants that appear healthier and are studier [27, 144]. The shorter and more compact plants resulting from mechanical stimulation not only improve aesthetic appeal but also improve volume-use efficiency, which is an important aspect in vertical farming systems [145]. Innovations in scaling up mechanical stimulation in the horticultural industry include directed air stream systems that have shown comparable results to stimulation by traditional touch-based methods [18].

The application of mechanical stimulation for field-grown crops, including cereals, is not new. A classic example is the practice of “mugifumi,” a process whereby mechanical stress is applied to wheat and barley seedlings through treading on them or using a roller [146]. This centuries-old Japanese tradition has shown the potential to increase yields and improve lodging resistance; however, its effectiveness depends on factors such as timing, frequency, genotype, and soil conditions, all of which require careful consideration [146].

Another example is the practice of co-cultivating rice and ducks in paddy fields, a technique with origins dating back over 400 years to China. Initially developed to combat grasshopper infestations in rice fields, this integrated farming approach not only provides economic and ecological benefits, but also a notable enhancement in rice productivity [147, 148]. Interestingly, it has been suggested that at least part of the beneficial effects of rice-duck farming systems on rice yield may be attributed to the mechanical stimulation exerted by ducks. This stimulation is believed to result in reduced plant height, increased stem diameter, a higher root/shoot ratio, and improved lodging resistance [149, 150].

**Fig. 4:**
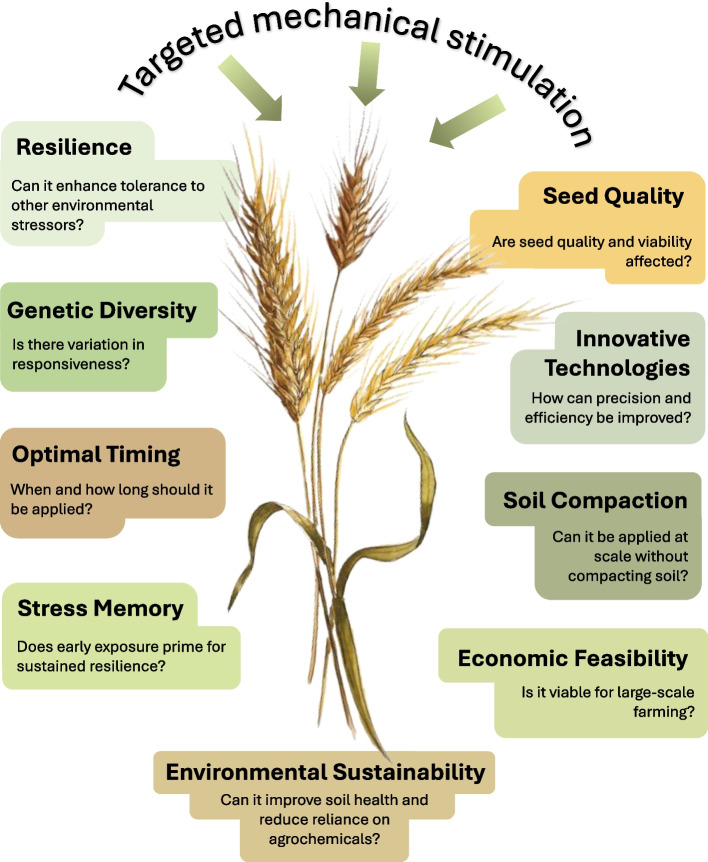
Challenges and future directions of targeted mechanical stimulation for agriculture. Key considerations for implementing targeted mechanical stimulation in agricultural systems are highlighted, focusing on optimizing resilience, genetic diversity, seed quality, and sustainability. It also addresses practical aspects such as timing, technology, soil health, and economic feasibility, aiming to balance precision with large-scale applicability

While techniques like mugifumi typically involve repeated mechanical stress over a certain period of time, it has also been shown that a single mechanical stimulation of wheat plants using a roller made them more resistant to cold stress [143]. In addition, this treatment, which also induced a level of mechanical wounding, decreased plant height, increased the lignin content, and enhanced the mechanical strength and lodging resistance post-anthesis, with no impact on yield [143]. These findings suggest that a singular mechanical stimulus can already prime plants to become more resilient when exposed to subsequent environmental stresses, offering the potential to develop novel management strategies for the production of wheat, other cereals, and grass crops (Fig. 4).

Mechanical stimulation can also affect neighboring plants that are not directly stimulated. For instance, brushing young maize plants induced the production and release of volatile organic compounds, activating defense genes in adjacent maize plants that were not stimulated by brushing [151]. Moreover, it is not only direct physical contact that can benefit grass crops; vibration-treatment of seeds, at 70 Hz, for instance, increased field germination rates, tiller numbers, and grain weight per plant in both a winter and spring wheat cultivar [152]. These findings suggest that mechanical seed priming is an additional avenue for exploration in optimizing crop performance.

As previously highlighted, lodging poses a significant threat to both the yield and quality of cereal crops, particularly during the reproductive stages. While mechanical stimulation has been proposed as a strategy to mitigate lodging risk in cereals, comprehensive studies on this subject are limited. A study comparing free-standing and supported wheat plants showed that free-standing plants produced more roots, resulting in a more robust root system with increased anchorage strength [153]. Beforementioned investigations in *Brachypodium* showed that mechanical stimulation resulted in the formation of more adventitious roots and induced cell wall thickening in root cortical sclerenchyma cells, thereby increasing resistance to lodging [92, 93]. The identification of SECONDARY WALL NAC7 as a putative regulator of the cell wall thickening response [93] provides scope for the genetic manipulation of lodging resistance. Overall, it is evident that above-ground mechanical stimulation induced by wind or otherwise, profoundly affects below-ground root growth and architecture. Changes to root systems induced by mechanical stimulation may have implications beyond increasing anchoring strength. Plant root systems are important for soil health and contribute to soil organic carbon mainly through root litter and rhizodeposition, including root exudates [154, 155]. It will be interesting to see if increases in root biomass and alterations in root architecture induced by mechanical stimulation have the potential to increase carbon inputs in the soil and improve soil health-related parameters more generally (Fig. 4). However, agricultural management practices aimed at improving crops through mechanical stimulation need to consider that the implementation of heavy machinery (e.g., tractors with rollers) may aggravate the problem of soil compaction which reduces root growth and therefore resource access [156] (Fig. 4). Notably, soil compaction itself induces mechanical stimulation to the root system, activating seemingly similar signaling pathways as those triggered by mechanical stimulation to the shoots [67].

Plant height plays a crucial role in stem lodging susceptibility. The use of semi-dwarfing genes, such as *Rht* in wheat and *sd-1* in rice, mostly interfering with the action or production of gibberellin, was crucial for the Green Revolution as it reduced plant height and increased yield [157]. Despite the remarkable productivity gains achieved through the adoption of dwarfing genes, there are notable environmental trade-offs. The substantial yield improvements of modern varieties can only be achieved by the intensive use of fertilizers. As a result, the use of inorganic fertilizers has increased tenfold since the Green Revolution [158]. Moreover, modern semi-dwarf varieties are typically less drought tolerant compared to landraces [159, 160]. Since giving young wheat plants a dose of mechanical stimulation decreases their height [17], and potentially increases root biomass, root anchorage, and overall plant resilience to environmental stresses, there is scope for mechanical stimulation to improve lodging resistance, as corroborated by previously mentioned studies on mechanical stimulation improving lodging tolerance [143, 146], and overall crop resilience while reducing reliance on agrochemical inputs (Fig. 4).

As previously mentioned, the mechanical properties of stem tissues, largely determined by the cell wall composition, represent another key determinant that affects stem lodging. For instance, several studies have shown that lodging-resistant varieties typically exhibit a higher lignin content [117, 123, 161]. Besides reducing plant height, mechanical stimulation of *Brachypodium* increased both stem lignin content and the rigidity of stem segments [16], providing further scope for improving lodging resistance through mechanical conditioning. Interestingly, mechanical stimulation has been shown to substantially increase the activity of the cell wall remodeling enzyme pectin methylesterase (PME) in *Brachypodium* stems and leaves [16], suggesting a higher proportion of rigid de-methylesterified pectins in the cell wall, which may contribute to altering the mechanical properties. The methylesterification status of cell wall pectins has been linked to resistance against various biotic and abiotic stresses [162–164]. It remains to be seen if the increase in PME activity induced by mechanical stimulation contributes to an improved resistance of stimulated crops against biotic and abiotic stresses.

The foregoing discussion underscores the potential of mechanical stimulation as a promising approach to improve the resilience of crops, particularly cereals, against environmental challenges. Plant priming is the phenomenon whereby the transient exposure to a stimulus elicits an enhanced defense response when exposed to future environmental stress conditions. While many priming methods rely on chemical agents, predominantly phytohormone-related metabolites, their usage raises environmental concerns, contributing to pollution and adversely impacting plant ecosystems and microbiota, consequently affecting soil fertility and crop yield [165]. In contrast, priming through mechanical stimulation, also known as thigmopriming, represents a promising alternative to chemical priming, offering an approach for the transition from traditional chemical agriculture to sustainable ecological farming practices.

Priming induces stress memory in plants, enabling them to respond more rapidly and robustly to subsequent environmental challenges. The duration of this memory depends, among other factors, on the exposure time and intensity of the priming stimulus [166]. Stress memory in plants occurs predominantly as transcriptional memory and epigenetic memory [166, 167]. Although it has been suggested that mechanical stimulation may induce these types of memory [25], future work will need to establish if mechanical stimulation at the seedling stage indeed induces stress memory, leading to improved resilience later, when plants experience challenging environmental conditions (Fig. 4). Alternatively, it remains to be determined if the morphological, developmental, and structural adaptations triggered by mechanical stimulation lead to improved resilience

## Conclusions and future perspectives

In conclusion, the potential of mechanical stimulation to enhance crop resilience is evident, yet several crucial questions remain unanswered. Firstly, it is essential to recognize that mechanical priming is not a “one size fits all” tool, and approaches may therefore need to be tailored for different species, cultivars, and local environments. Moreover, considering the context of critical height and dwarfing genes, further research is needed to determine if additional height reduction through mechanical priming is feasible without compromising plant stature beyond critical levels. However, mechanical stimulation offers opportunities to be combined with the exploration of wider germplasm diversity for agricultural implementation. Furthermore, while there is a growing understanding of how mechanical stimulation impacts aboveground yield components, its effect on the grain quality of cereals and on root systems remains poorly understood. Future work should aim to clarify whether priming induced by mechanical stimulation primarily involves morphological and developmental adaptations or includes transcriptional memory and epigenetic mechanisms. Additionally, the duration of priming-induced memory remains to be established (Fig. 4). Lastly, the molecular mechanism linking the perception of mechanical stimuli with the regulation of the responsive gene network that leads to subsequent morphogenetic adaptations and stress memory is almost completely uncharacterized in cereals. Thus, while the potential of mechanical stimulation in agriculture is promising, further research is necessary to unlock its full benefits and address remaining uncertainties.

## Data Availability

Not applicable

## References

[1] Jaffe MJ. Thigmomorphogenesis: the response of plant growth and development to mechanical stimulation: with special reference to *Bryonia dioica*. Planta. 1973;114:143–57. 24458719 10.1007/BF00387472

[2] Jaffe MJ. Morphogenetic responses of plants to mechanical stimuli or stress. Bioscience. 1980;30(4):239–43.

[3] Hamant O. Widespread mechanosensing controls the structure behind the architecture in plants. Curr Opin Plant Biol. 2013;16(5):654–60. 23830994 10.1016/j.pbi.2013.06.006

[4] Codjoe JM, Miller K, Haswell ES. Plant cell mechanobiology: Greater than the sum of its parts. Plant Cell. 2022;34(1):129–45. 34524447 10.1093/plcell/koab230 PMC8773992

[5] Du F, Jiao Y. Mechanical control of plant morphogenesis: concepts and progress. Curr Opin Plant Biol. 2020;57:16–23. 32619966 10.1016/j.pbi.2020.05.008

[6] Hamant O, Saunders TE. Shaping organs: shared structural principles across kingdoms. Annu Rev Cell Dev Biol. 2020;36:385–410. 32628862 10.1146/annurev-cellbio-012820-103850

[7] Robinson S. Mechanobiology of cell division in plant growth. New Phytol. 2021;231(2):559–64. 33774836 10.1111/nph.17369

[8] Sampathkumar A. Mechanical feedback-loop regulation of morphogenesis in plants. Development. 2020;147(16):dev177964. 32817056 10.1242/dev.177964

[9] Einarson B, Link GKK: Theophrastus, De causis plantarum. London and Cambridge, Mass.: W Heinemann and Harvard University Press; 1976.

[10] Liu Z, Fadiji T, Yang J, Li Z, Tchuenbou-Magaia F. Impact of mechanical stimulation on the life cycle of horticultural plant. Horticultural Plant Journal. 2023;9(3):381–94.

[11] Biddington NL. The effects of mechanically-induced stress in plants - a review. Plant Growth Regul. 1986;4:103–23.

[12] Zhang S, Liu G, Cui Q, Huang Z, Ye X, Cornelissen JHC. New field wind manipulation methodology reveals adaptive responses of steppe plants to increased and reduced wind speed. Plant Methods. 2021;17(1):5. 33407697 10.1186/s13007-020-00705-2 PMC7788872

[13] Baden SA, Latimer JG. An effective system for brushing vegetable transplants for height control. HortTechnology. 1992;2(3):412–4.

[14] Chehab EW, Eich E, Braam J. Thigmomorphogenesis: a complex plant response to mechano-stimulation. J Exp Bot. 2009;60(1):43–56. 19088336 10.1093/jxb/ern315

[15] Erner Y, Jaffe MJ. Thigmomorphogenesis: The involvement of auxin and abscisic acid in growth retardation due to mechanical perturbation. Plant Cell Physiol. 1982;23(6):935–41.

[16] Gladala-Kostarz A, Doonan JH, Bosch M. Mechanical stimulation in *Brachypodium distachyon*: Implications for fitness, productivity, and cell wall properties. Plant Cell Environ. 2020;43(5):1314–30. 31955437 10.1111/pce.13724 PMC7318644

[17] Hindhaugh R, Bosch M, Donnison IS. Mechanical stimulation in wheat triggers age- and dose-dependent alterations in growth, development and grain characteristics. Ann Bot. 2021;128(5):589–603. 34091667 10.1093/aob/mcab070 PMC8422892

[18] Sparke M-A, Wegscheider A, Winterhagen P, Ruttensperger U, Hegele M, Wünsche JN. Air-based mechanical stimulation controls plant height of ornamental plants and vegetable crops under greenhouse conditions. HortTechnology. 2021;31(4):405–16.

[19] Telewski FW. Mechanosensing and plant growth regulators elicited during the thigmomorphogenetic response. Front For Glob Change. 2021;3:574096.

[20] Braam J, Davis RW. Rain-, wind-, and touch-induced expression of calmodulin and calmodulin-related genes in Arabidopsis. Cell. 1990;60:357–64. 2302732 10.1016/0092-8674(90)90587-5

[21] Seneviratne SI, Zhang X, Adnan M, Badi W, Dereczynski C, Di Luca A, Ghosh S, Iskandar I, Kossin J, Lewis S et al*.* 2021: Weather and climate extreme events in a changing climate. In Climate Change 2021: The physical science basis. Contribution of working group I to the sixth assessment report of the intergovernmental panel on climate change. 2021 (pp. 1513–1766). Cambridge University Press, Cambridge, United Kingdom and New York, NY, USA.

[22] Seeburger P, Herdenstam A, Kurtser P, Arunachalam A, Castro-Alves VC, Hyotylainen T, Andreasson H. Controlled mechanical stimuli reveal novel associations between basil metabolism and sensory quality. Food Chem. 2023;404:134545. 36252376 10.1016/j.foodchem.2022.134545

[23] Šic Žlabur J, Radman S, Fabek Uher S, Opačić N, Benko B, Galić A, Samirić P, Voća S. Plant response to mechanically-induced stress: a case study on specialized metabolites of leafy vegetables. Plants. 2021;10(12): 2650. 34961120 10.3390/plants10122650 PMC8709336

[24] Li Q, Zargar O, Park S, Pharr M, Muliana A, Finlayson SA. Mechanical stimulation reprograms the sorghum internode transcriptome and broadly alters hormone homeostasis. Plant Sci. 2023;327:111555. 36481363 10.1016/j.plantsci.2022.111555

[25] Brenya E, Pervin M, Chen ZH, Tissue DT, Johnson S, Braam J, Cazzonelli CI. Mechanical stress acclimation in plants: Linking hormones and somatic memory to thigmomorphogenesis. Plant Cell Environ. 2022;45(4):989–1010. 34984703 10.1111/pce.14252

[26] Kouhen M, Dimitrova A, Scippa GS, Trupiano D. The course of mechanical stress: types, perception, and plant response. Biology. 2023;12(2): 217. 36829495 10.3390/biology12020217 PMC9953051

[27] Börnke F, Rocksch T. Thigmomorphogenesis – Control of plant growth by mechanical stimulation. Sci Hortic. 2018;234:344–53.

[28] Sparke MA, Wünsche JN. Mechanosensing of plants. Hortic Rev. 2020;47:43–83.

[29] Darwish E, Ghosh R, Ontiveros-Cisneros A, Tran HC, Petersson M, De Milde L, Broda M, Goossens A, Van Moerkercke A, Khan K, Van Aken O. Touch signaling and thigmomorphogenesis are regulated by complementary CAMTA3-and JA-dependent pathways. Science Advances. 2022;8(20):eabm2091. 35594358 10.1126/sciadv.abm2091 PMC9122320

[30] Hamant O, Inoue D, Bouchez D, Dumais J, Mjolsness E. Are microtubules tension sensors? Nat Commun. 2019;10(1):2360. 31142740 10.1038/s41467-019-10207-y PMC6541610

[31] Shevchenko GV, Krutovsky KV. Mechanical stress effects on transcriptional regulation of genes encoding microtubule- and actin-associated proteins. Physiol Mol Biol Plants. 2022;28(1):17–30. 35210715 10.1007/s12298-021-01123-x PMC8847523

[32] Hamant O, Haswell ES. Life behind the wall: sensing mechanical cues in plants. BMC Biol. 2017;15(1):59. 28697754 10.1186/s12915-017-0403-5 PMC5505048

[33] Monshausen GB, Haswell ES. A force of nature: molecular mechanisms of mechanoperception in plants. J Exp Bot. 2013;64(15):4663–80. 23913953 10.1093/jxb/ert204 PMC3817949

[34] Shih HW, Miller ND, Dai C, Spalding EP, Monshausen GB. The receptor-like kinase FERONIA is required for mechanical signal transduction in Arabidopsis seedlings. Curr Biol. 2014;24(16):1887–92. 25127214 10.1016/j.cub.2014.06.064

[35] Feng W, Kita D, Peaucelle A, Cartwright HN, Doan V, Duan Q, Liu MC, Maman J, Steinhorst L, Schmitz-Thom I, Yvon R. The FERONIA receptor kinase maintains cell-wall integrity during salt stress through Ca^2+^ signaling. Curr Biol. 2018;28(5):666–75. 29456142 10.1016/j.cub.2018.01.023 PMC5894116

[36] Galindo-Trigo S, Gray JE, Smith LM. Conserved roles of CrRLK1L receptor-like kinases in cell expansion and reproduction from algae to angiosperms. Front Plant Sci. 2016;7:1269. 27621737 10.3389/fpls.2016.01269 PMC5002434

[37] Van der Does D, Boutrot F, Engelsdorf T, Rhodes J, McKenna JF, Vernhettes S, Koevoets I, Tintor N, Veerabagu M, Miedes E, et al. The Arabidopsis leucine-rich repeat receptor kinase MIK2/LRR-KISS connects cell wall integrity sensing, root growth and response to abiotic and biotic stresses. PLoS Genet. 2017;13(6):e1006832. 28604776 10.1371/journal.pgen.1006832 PMC5484538

[38] Wolf S, van der Does D, Ladwig F, Sticht C, Kolbeck A, Schurholz AK, Augustin S, Keinath N, Rausch T, Greiner S, Schumacher K. A receptor-like protein mediates the response to pectin modification by activating brassinosteroid signaling. Proc Natl Acad Sci U S A. 2014;111(42):15261–6. 25288746 10.1073/pnas.1322979111 PMC4210321

[39] Xu SL, Rahman A, Baskin TI, Kieber JJ. Two leucine-rich repeat receptor kinases mediate signaling, linking cell wall biosynthesis and ACC synthase in Arabidopsis. Plant Cell. 2008;20(11):3065–79. 19017745 10.1105/tpc.108.063354 PMC2613664

[40] Knight MR, Smith SM, Trewavas AJ. Wind-induced plant motion immdediately increases cytosolic calcium. Proc Natl Acad Sci U S A. 1992;89:4967–71. 11536497 10.1073/pnas.89.11.4967 PMC49209

[41] Botella JR, Arteca JM, Somodevilla M, Arteca RN. Calcium-dependent protein kinase gene expression in response to physical and chemical stimuli in mungbean (*Vigna radiata*). Plant Mol Biol. 1996;30:1129–37. 8704124 10.1007/BF00019547

[42] Hashimoto K, Kudla J. Calcium decoding mechanisms in plants. Biochimie. 2011;93(12):2054–9. 21658427 10.1016/j.biochi.2011.05.019

[43] Benikhlef L, L’Haridon FL, Abou-Mansour E, Serrano M, Binda M, Costa A, Lehmann S, Metraux J-P. Perception of soft mechanical stress in Arabidopsis leaves activates disease resistance. BMC Plant Biol. 2013;13:133. 24033927 10.1186/1471-2229-13-133 PMC3848705

[44] Depège N, Varenne M, Boyer N. Induction of oxidative stress and GPX-like protein activation in tomato plants after mechanical stimulation. Physiol Plant. 2000;110(2):209–14.

[45] Herbette S, de Labrouhe DT, Drevet JR, Roeckel-Drevet P. Transgenic tomatoes showing higher glutathione peroxydase antioxidant activity are more resistant to an abiotic stress but more susceptible to biotic stresses. Plant Sci. 2011;180(3):548–53. 21421403 10.1016/j.plantsci.2010.12.002

[46] Mori IC, Schroeder JI. Reactive oxygen species activation of plant Ca2+ channels. A signaling mechanism in polar growth, hormone transduction, stress signaling, and hypothetically mechanotransduction. Plant physiology. 2004;135(2):702–8. 15208417 10.1104/pp.104.042069 PMC514107

[47] Monshausen GB, Bibikova TN, Weisenseel MH, Gilroy S. Ca^2+^ regulates reactive oxygen species production and pH during mechanosensing in Arabidopsis roots. Plant Cell. 2009;21(8):2341–56. 19654264 10.1105/tpc.109.068395 PMC2751959

[48] Behera S, Zhaolong X, Luoni L, Bonza MC, Doccula FG, De Michelis MI, Morris RJ, Schwarzlander M, Costa A. Cellular Ca^2+^ signals generate defined pH signatures in plants. Plant Cell. 2018;30(11):2704–19. 30377237 10.1105/tpc.18.00655 PMC6305977

[49] Bosch M, Franklin-Tong V. Regulating programmed cell death in plant cells: Intracellular acidification plays a pivotal role together with calcium signaling. Plant Cell. 2024;36(11):4692–702. 10.1093/plcell/koae245 PMC11530775 39197046

[50] Lee D, Polisensky DH, Braam J. Genome-wide identification of touch- and darkness-regulated Arabidopsis genes: a focus on calmodulin-like and XTH genes. New Phytol. 2005;165(2):429–44. 15720654 10.1111/j.1469-8137.2004.01238.x

[51] Pomiès L, Decourteix M, Franchel J, Moulia B, Leblanc-Fournier N. Poplar stem transcriptome is massively remodelled in response to single or repeated mechanical stimuli. BMC Genomics. 2017;18:300. 28412928 10.1186/s12864-017-3670-1 PMC5392906

[52] Braam J. In touch: plant responses to mechanical stimuli. New Phytol. 2005;165(2):373–89. 15720650 10.1111/j.1469-8137.2004.01263.x

[53] Fluch S, Olmo CC, Tauber S, Stierschneider M, Kopecky D, Reichenauer TG, Matusikova I. Transcriptomic changes in wind-exposed poplar leaves are dependent on developmental stage. Planta. 2008;228(5):757–64. 18719940 10.1007/s00425-008-0777-2

[54] Wang K, Yang Z, Qing D, Ren F, Liu S, Zheng Q, Liu J, Zhang W, Dai C, Wu M, et al. Quantitative and functional posttranslational modification proteomics reveals that TREPH1 plays a role in plant touch-delayed bolting. Proc Natl Acad Sci U S A. 2018;115(43):E10265–74. 30291188 10.1073/pnas.1814006115 PMC6205429

[55] Stelmach BA, Müller A, Hennig P, Laudert D, Andert L, Weiler EW. Quantitation of the octadecanoid 12-oxo-phytodienoic acid, a signalling compound in plant mechanotransduction. Phytochemistry. 1998;47(4):539–46. 9461672 10.1016/s0031-9422(97)00547-5

[56] Chehab EW, Yao C, Henderson Z, Kim S, Braam J. Arabidopsis touch-induced morphogenesis is jasmonate mediated and protects against pests. Curr Biol. 2012;22(8):701–6. 22483939 10.1016/j.cub.2012.02.061

[57] Van Moerkercke A, Duncan O, Zander M, Simura J, Broda M, Vanden Bossche R, Lewsey MG, Lama S, Singh KB, Ljung K, Ecker JR. A MYC2/MYC3/MYC4-dependent transcription factor network regulates water spray-responsive gene expression and jasmonate levels. Proc Natl Acad Sci U S A. 2019;116(46):23345–56. 31662474 10.1073/pnas.1911758116 PMC6859355

[58] Johns S, Hagihara T, Toyota M, Gilroy S. The fast and the furious: rapid long-range signaling in plants. Plant Physiol. 2021;185(3):694–706. 33793939 10.1093/plphys/kiaa098 PMC8133610

[59] Yan C, Fan M, Yang M, Zhao J, Zhang W, Su Y, Xiao L, Deng H, Xie D. Injury activates Ca^2+^/calmodulin-dependent phosphorylation of JAV1-JAZ8-WRKY51 complex for jasmonate biosynthesis. Mol Cell. 2018;70(1):136–49. 29625034 10.1016/j.molcel.2018.03.013

[60] Takahashi H, Suge H. Sex expression in cucumber plants as affected by mechanical stress. Plant Cell Physiol. 1980;21(2):303–10.

[61] Lange MJ, Lange T. Touch-induced changes in Arabidopsis morphology dependent on gibberellin breakdown. Nat Plants. 2015;1:14025. 27246879 10.1038/nplants.2014.25

[62] Johnson KA, Sistrunk ML, Polisensky DH, Braam J. *Arabidopsis thaliana* responses to mechanical stimulation do not require ETR1 or EIN2. Plant Physiol. 1998;116(2):643–9. 9489014 10.1104/pp.116.2.643 PMC35122

[63] Biro R, Jaffe MJ. Thigmomorphogenesis: ethylene evolution and its role in the changes observed in mechanically perturbed bean plants. Physiol Plant. 1984;62(3):289–96. 10.1111/j.1399-3054.1984.tb05925.x 11540788

[64] Goeschl JD, Rappaport L, Pratt HK. Ethylene as a factor regulating the growth of pea epicotyls subjected to physical stress. Plant Physiol. 1966;41(5):877–84. 16656334 10.1104/pp.41.5.877 PMC1086440

[65] Arteca JM, Arteca RN. A multi-responsive gene encoding 1-aminocyclopropane-1-carboxylate synthase (ACS6) in mature Arabidopsis leaves. Plant Mol Biol. 1999;39:209–19. 10080689 10.1023/a:1006177902093

[66] Botella JR, Arteca RN, Frangos JA. A mechanical strain-induced 1-aminocyclopropane-1-carboxylic acid synthase gene. Proc Natl Acad Sci U S A. 1995;92(5):1595–8. 7878024 10.1073/pnas.92.5.1595 PMC42566

[67] Anten NPR, Casado-Garcia R, Pierik R, Pons TL. Ethylene sensitivity affects changes in growth patterns, but not stem properties, in response to mechanical stress in tobacco. Physiol Plant. 2006;128(2):274–82.

[68] Wu Q, Li Y, Lyu M, Luo Y, Shi H, Zhong S. Touch-induced seedling morphological changes are determined by ethylene-regulated pectin degradation. Science advances. 2020;6(48):eabc9294. 33246960 10.1126/sciadv.abc9294 PMC7695475

[69] Wang L, Ma C, Wang S, Yang F, Sun Y, Tang J, Luo J, Wu J. Ethylene and jasmonate signaling converge on gibberellin catabolism during thigmomorphogenesis in Arabidopsis. Plant Physiol. 2024;194(2):758–73. 37847103 10.1093/plphys/kiad556

[70] Xu W, Purugganan MM, Polisensky DH, Antosiewicz DM, Fry SC, Braam J. Arabidopsis TCH4, regulated by hormones and the environment, encodes a xyloglucan endotransglycosylase. Plant Cell. 1995;7(10):1555–67. 7580251 10.1105/tpc.7.10.1555 PMC161010

[71] Awika JM. Major cereal grains production and use around the world. In: Advances in cereal science: implications to food processing and health promotion. ACS Publications; 2011. p. 1–13. 10.1021/bk-2011-1089.ch001.

[72] Brosnan JT, Ebdon J, Dest W. Characteristics in diverse wear tolerant genotypes of Kentucky bluegrass. Crop Sci. 2005;45(5):1917–26.

[73] Głąb T, Szewczyk W, Kopeć-Jarosz A. Comprehensive assessment of turfgrass wear tolerance: A study on mechanical and chemical traits. Ind Crops Prod. 2024;216:118837.

[74] Darwish E, Ghosh R, Bentzer J, Tsardakas Renhuldt N, Proux-Wera E, Kamal N, Spannagl M, Hause B, Sirijovski N, Van Aken O. The dynamics of touch-responsive gene expression in cereals. Plant J. 2023;116(1):282–302. 37159480 10.1111/tpj.16269

[75] Markovic D, Glinwood R, Olsson U, Ninkovic V. Plant response to touch affects the behaviour of aphids and ladybirds. Arthropod-Plant Interactions. 2014;8:171–81.

[76] Steucek G, Gordon L. Response of wheat (*Triticum aestivum*) seedlings to mechanical stress. Bot Gaz. 1975;136(1):17–9.

[77] Zargar O, Li Q, Nwaobi C, Pharr M, Finlayson SA, Muliana A. Thigmostimulation alters anatomical and biomechanical properties of bioenergy sorghum stems. J Mech Behav Biomed Mater. 2022;127: 105090. 35114492 10.1016/j.jmbbm.2022.105090

[78] Zhao B, Teng L, Zhang J-E, Xiang H, Li M, Liang K. Thigmotropic responses of Oryza sativa L. to external rubbing stimulation. Archives of Biological Sciences. 2018;70(1):129–39.

[79] Goodman A, Ennos A. A comparative study of the response of the roots and shoots of sunflower and maize to mechanical stimulation. J Exp Bot. 1996;47(10):1499–507.

[80] Jellum M. Relationships between lodging resistance and certain culm characters in oats. Crop Sci. 1962;2:263–7.

[81] Grace J, Russell G. The effect of wind on grasses: III. Influence of continuous drought or wind on anatomy and water relations in festuca arundinacea schreb. Journal of Experimental Botany. 1977;28(2):268–78.

[82] Zhang J-E, Quan G-M, Huang Z-X, Luo S-M, Ouyang Y. Evidence of duck activity induced anatomical structure change and lodging resistance of rice plant. Agroecol Sustain Food Syst. 2013;37(9):975–84.

[83] da Costa RM, Pattathil S, Avci U, Lee SJ, Hazen SP, Winters A, Hahn MG, Bosch M. A cell wall reference profile for *Miscanthus* bioenergy crops highlights compositional and structural variations associated with development and organ origin. New Phytol. 2017;213(4):1710–25. 27859277 10.1111/nph.14306 PMC5324610

[84] Bhatia R, Timms-Taravella E, Roberts LA, Moron-Garcia OM, Hauck B, Dalton S, Gallagher JA, Wagner M, Clifton-Brown J, Bosch M. Transgenic ZmMYB167 *Miscanthus sinensis* with increased lignin to boost bioenergy generation for the bioeconomy. Biotechnology for Biofuels and Bioproducts. 2023;16(1):29. 36814294 10.1186/s13068-023-02279-2 PMC9945411

[85] Bhatia R, Gallagher JA, Gomez LD, Bosch M. Genetic engineering of grass cell wall polysaccharides for biorefining. Plant Biotechnol J. 2017;15(9):1071–92. 28557198 10.1111/pbi.12764 PMC5552484

[86] Cornwall J, Stubbs CJ, McMahan CS, Robertson DJ. The overlooked biomechanical role of the clasping leaf sheath in wheat stalk lodging. Front Plant Sci. 2021;12:617880. 34489984 10.3389/fpls.2021.617880 PMC8417718

[87] Gomez FE, Muliana AH, Niklas KJ, Rooney WL. Identifying morphological and mechanical traits associated with stem lodging in bioenergy sorghum (*Sorghum bicolor*). BioEnergy Research. 2017;10:635–47.

[88] Ookawa T, Hobo T, Yano M, Murata K, Ando T, Miura H, Asano K, Ochiai Y, Ikeda M, Nishitani R. New approach for rice improvement using a pleiotropic QTL gene for lodging resistance and yield. Nat Commun. 2010;1(1): 132. 21119645 10.1038/ncomms1132 PMC3065348

[89] Robertson DJ, Julias M, Gardunia BW, Barten T, Cook DD. Corn stalk lodging: a forensic engineering approach provides insights into failure patterns and mechanisms. Crop Sci. 2015;55(6):2833–41.

[90] Goodman A, Ennos A. The responses of field-grown sunflower and maize to mechanical support. Ann Bot. 1997;79(6):703–11.

[91] Wang YH, Yu FH, Dong M, Lin XQ, Jiang H, He WM: Growth and biomass allocation of Lolium perenne seedlings in response to mechanical stimulation and water availability. In: Annales Botanici Fennici: 2010. (Vol. 47, No. 5, pp. 367–372). Finnish Zoological and Botanical Publishing Board.

[92] Nam BE, Park YJ, Gil KE, Kim JH, Kim JG, Park CM. Auxin mediates the touch-induced mechanical stimulation of adventitious root formation under windy conditions in *Brachypodium distachyon*. BMC Plant Biol. 2020;20(1):335. 32678030 10.1186/s12870-020-02544-8 PMC7364541

[93] McCahill IW, Khahani B, Probert CF, Flockhart EL, Abushal LT, Gregory GA, Zhang Y, Baumgart LA, O'Malley RC, Hazen SP. Shoring up the base: the development and regulation of cortical sclerenchyma in grass nodal roots. bioRxiv. 2024.01.25.577257. 10.1093/plphys/kiaf215 40460244

[94] Chen YL, Palta J, Clements J, Buirchell B, Siddique KH, Rengel Z. Root architecture alteration of narrow-leafed lupin and wheat in response to soil compaction. Field Crop Res. 2014;165:61–70.

[95] Colombi T, Braun S, Keller T, Walter A. Artificial macropores attract crop roots and enhance plant productivity on compacted soils. Sci Total Environ. 2017;574:1283–93. 27712865 10.1016/j.scitotenv.2016.07.194

[96] Pandey BK, Huang G, Bhosale R, Hartman S, Sturrock CJ, Jose L, Martin OC, Karady M, Voesenek LA, Ljung K. Plant roots sense soil compaction through restricted ethylene diffusion. Science. 2021;371(6526):276–80. 33446554 10.1126/science.abf3013

[97] Atwell B. The effect of soil compaction on wheat during early tillering: III. Fate of carbon transported to the roots. New Phytologist. 1990;115(1):43–9.

[98] Iijima M, Kono Y, Yamauchi A, Pardales J Jr. Effects of soil compaction on the development of rice and maize root systems. Environ Exp Bot. 1991;31(3):333–42.

[99] Lipiec J, Horn R, Pietrusiewicz J, Siczek A. Effects of soil compaction on root elongation and anatomy of different cereal plant species. Soil and Tillage Research. 2012;121:74–81.

[100] Loades K, Bengough A, Bransby M, Hallett P. Biomechanics of nodal, seminal and lateral roots of barley: effects of diameter, waterlogging and mechanical impedance. Plant Soil. 2013;370:407–18.

[101] Potocka I, Szymanowska-Pułka J. Morphological responses of plant roots to mechanical stress. Ann Bot. 2018;122(5):711–23. 29471488 10.1093/aob/mcy010 PMC6215033

[102] Handakumbura PP, Hazen SP. Transcriptional regulation of grass secondary cell wall biosynthesis: playing catch-up with *Arabidopsis thaliana*. Front Plant Sci. 2012;3:74. 22639662 10.3389/fpls.2012.00074 PMC3355686

[103] Albrecht T, Kehlen A, Stahl K, Knöfel H-D, Sembdner G, Weiler E. Quantification of rapid, transient increases in jasmonic acid in wounded plants using a monoclonal antibody. Planta. 1993;191(1):86–94.

[104] Mauch F, Kmecl A, Schaffrath U, Volrath S, Gorlach J, Ward E, Ryals J, Dudler R. Mechanosensitive expression of a lipoxygenase gene in wheat. Plant Physiol. 1997;114(4):1561–6. 9276964 10.1104/pp.114.4.1561 PMC158451

[105] Coomey JH, MacKinnon KJ-M, McCahill IW, Khahani B, Handakumbura PP, Trabucco GM, Mazzola J, Leblanc NA, Kheam R, Hernandez-Romero M. Mechanically induced localisation of SECONDARY WALL INTERACTING bZIP is associated with thigmomorphogenic and secondary cell wall gene expression. Quantitative. Plant Biology. 2024;5:e5. 10.1017/qpb.2024.5 PMC11106548 38774130

[106] Pinthus MJ. Lodging in wheat, barley, and oats: the phenomenon, its causes, and preventive measures. Adv Agron. 1974;25:209–63.

[107] Berry P, Sterling M, Spink J, Baker C, Sylvester-Bradley R, Mooney S, Tams A, Ennos A. Understanding and reducing lodging in cereals. Adv Agron. 2004;84(04):215–69.

[108] Sterling M, Baker CJ, Berry PM, Wade A. An experimental investigation of the lodging of wheat. Agric For Meteorol. 2003;119(3–4):149–65.

[109] Berry P, Spink J. Predicting yield losses caused by lodging in wheat. Field Crop Res. 2012;137:19–26.

[110] Flint-Garcia SA, Jampatong C, Darrah LL, McMullen MD. Quantitative trait locus analysis of stalk strength in four maize populations. Crop Sci. 2003;43(1):13–22.

[111] Berry PM, Sterling M, Mooney SJ. Development of a model of lodging for Barley. J Agron Crop Sci. 2006;192(2):151–8.

[112] Setter T, Laureles E, Mazaredo A. Lodging reduces yield of rice by self-shading and reductions in canopy photosynthesis. Field Crop Res. 1997;49(2–3):95–106.

[113] Gornall J, Betts R, Burke E, Clark R, Camp J, Willett K, Wiltshire A. Implications of climate change for agricultural productivity in the early twenty-first century. Philosophical Transactions of the Royal Society B: Biological Sciences. 2010;365(1554):2973–89. 10.1098/rstb.2010.0158 PMC2935125 20713397

[114] Chauhan S, Darvishzadeh R, Boschetti M, Pepe M, Nelson A. Remote sensing-based crop lodging assessment: Current status and perspectives. ISPRS J Photogramm Remote Sens. 2019;151:124–40.

[115] Zhang Z, Flores P, Igathinathane CL, Naik D, Kiran R, Ransom JK. Wheat lodging detection from UAS imagery using machine learning algorithms. Remote sensing. 2020;12(11):1838.

[116] Shah AN, Tanveer M, Rehman AU, Anjum SA, Iqbal J, Ahmad R. Lodging stress in cereal—effects and management: an overview. Environ Sci Pollut Res. 2017;24:5222–37. 10.1007/s11356-016-8237-1 28025787

[117] Niu Y, Chen T, Zhao C, Zhou M. Improving crop lodging resistance by adjusting plant height and stem strength. Agronomy. 2021;11(12):2421.

[118] Peng Y, Hu Y, Qian Q, Ren D. Progress and prospect of breeding utilization of Green Revolution gene SD 1 in rice. Agriculture. 2021;11(7): 611.

[119] Niu Y, Chen T, Zhao C, Zhou M. Lodging prevention in cereals: Morphological, biochemical, anatomical traits and their molecular mechanisms, management and breeding strategies. Field Crops Research. 2022;289:108733.

[120] Khobra R, Sareen S, Meena BK, Kumar A, Tiwari V, Singh G. Exploring the traits for lodging tolerance in wheat genotypes: a review. Physiol Mol Biol Plants. 2019;25:589–600. 31168225 10.1007/s12298-018-0629-x PMC6522606

[121] Li Q, Fu C, Liang C, Ni X, Zhao X, Chen M, Ou L. Crop lodging and the roles of lignin, cellulose, and hemicellulose in lodging resistance. Agronomy. 2022;12(8):1795.

[122] Mengistie E, McDonald AG. Effect of cell wall compositions on lodging resistance of cereal crops: review. J Agric Sci. 2024;161(6):794–807.

[123] Peng D, Chen X, Yin Y, Lu K, Yang W, Tang Y, Wang Z. Lodging resistance of winter wheat (Triticum aestivum L.): Lignin accumulation and its related enzymes activities due to the application of paclobutrazol or gibberellin acid. Field Crops Research. 2014;157:1–7.

[124] Shah L, Yahya M, Shah SMA, Nadeem M, Ali A, Ali A, Wang J, Riaz MW, Rehman S, Wu W, Khan RM. Improving lodging resistance: using wheat and rice as classical examples. Int J Mol Sci. 2019;20(17): 4211. 31466256 10.3390/ijms20174211 PMC6747267

[125] Liu L, Liu S, Lu H, Tian Z, Zhao H, Wei D, Wang S, Huang Z. Integration of transcriptome and metabolome analyses reveals key lodging-resistance-related genes and metabolic pathways in maize. Front Genet. 2022;13: 1001195. 36299597 10.3389/fgene.2022.1001195 PMC9588961

[126] Muhammad A, Hao H, Xue Y, Alam A, Bai S, Hu W, Sajid M, Hu Z, Samad RA, Li Z, Liu P. Survey of wheat straw stem characteristics for enhanced resistance to lodging. Cellulose. 2020;27(5):2469–84.

[127] Lv S, Lin Z, Shen J, Luo L, Xu Q, Li L, Gui J. OsTCP19 coordinates inhibition of lignin biosynthesis and promotion of cellulose biosynthesis to modify lodging resistance in rice. J Exp Bot. 2024;75(1):123–36. 37724960 10.1093/jxb/erad367

[128] Li F, Xie G, Huang J, Zhang R, Li Y, Zhang M, Wang Y, Li A, Li X, Xia T, Qu C. OsCESA9 conserved-site mutation leads to largely enhanced plant lodging resistance and biomass enzymatic saccharification by reducing cellulose DP and crystallinity in rice. Plant Biotechnol J. 2017;15(9):1093–104. 28117552 10.1111/pbi.12700 PMC5552474

[129] Kong E, Liu D, Guo X, Yang W, Sun J, Li X, Zhan K, Cui D, Lin J, Zhang A. Anatomical and chemical characteristics associated with lodging resistance in wheat. The Crop Journal. 2013;1(1):43–9.

[130] Berry P, Griffin J, Sylvester-Bradley R, Scott R, Spink J, Baker C, Clare R. Controlling plant form through husbandry to minimise lodging in wheat. Field Crop Res. 2000;67(1):59–81.

[131] Berry P, Sylvester-Bradley R, Berry S. Ideotype design for lodging-resistant wheat. Euphytica. 2007;154(1):165–79.

[132] Dreccer MF, Condon AG, Macdonald B, Rebetzke GJ, Awasi M-A, Borgognone MG, Peake A, Piñera-Chavez FJ, Hundt A, Jackway P, McIntyre CL. Genotypic variation for lodging tolerance in spring wheat: wider and deeper root plates, a feature of low lodging, high yielding germplasm. Field Crops Research. 2020;258:107942.

[133] Terashima K. Eco-physiological study of root lodging tolerance in direct-seeded rice cultivars. Japan Agricultural Research Quarterly. 1997;31:155–62.

[134] Crook M, Ennos A. The effect of nitrogen and growth regulators on stem and root characteristics associated with lodging in two cultivars of winter wheat. J Exp Bot. 1995;46(8):931–8.

[135] Zhang W, Wu L, Ding Y, Yao X, Wu X, Weng F, Li G, Liu Z, Tang S, Ding C, Wang S. Nitrogen fertilizer application affects lodging resistance by altering secondary cell wall synthesis in japonica rice (*Oryza sativa*). J Plant Res. 2017;130(5):859–71. 28451936 10.1007/s10265-017-0943-3

[136] Zhang W-J, Wu L-M, Ding Y-F, Fei W, Wu X-R, Li G-H, Liu Z-H, She T, Ding C-Q, Wang S-H. Top-dressing nitrogen fertilizer rate contributes to decrease culm physical strength by reducing structural carbohydrate content in japonica rice. J Integr Agric. 2016;15(5):992–1004.

[137] Dorairaj D, Ismail MR, Sinniah UR, Kar BT. Influence of silicon on growth, yield, and lodging resistance of MR219, a lowland rice of Malaysia. J Plant Nutr. 2017;40(8):1111–24.

[138] Wang Y, Pan Y, Zhao F, Meng X, Li Q, Huang Y, Ye Y. Changes in the lodging resistance of winter wheat from 1950s to the 2020s in Henan Province of China. BMC Plant Biol. 2023;23(1):442. 37726651 10.1186/s12870-023-04452-z PMC10510142

[139] Liu J, Fan YF, Sun JY, Gao JL, Wang ZG, Yu XF. Effects of straw return with potassium fertilizer on the stem lodging resistance, grain quality and yield of spring maize (Zea mays L.). Sci Rep. 2023;13(1):20307. 37985725 10.1038/s41598-023-46569-z PMC10662436

[140] Zhang T, He X, Chen B, He L, Tang X. Effects of different potassium (K) fertilizer rates on yield formation and lodging of rice. Phyton. 2021;90(3):815–26.

[141] Clarke J, Wynn S, Twining S, Berry P, Cook S, Ellis S, Gladders P. Pesticide availability for cereals and oilseeds following revision of Directive 91/414/ECC; effects of losses and new research priorities. 2009. Research Review No. 70, Home Grown Cereals Authority:1-131 Boxworth, Cambridge. https://www.cabidigitallibrary.org/doi/full/10.5555/20093144654.

[142] Kamran M, Cui W, Ahmad I, Meng X, Zhang X, Su W, Chen J, Ahmad S, Fahad S, Han Q, Liu T. Effect of paclobutrazol, a potential growth regulator on stalk mechanical strength, lignin accumulation and its relation with lodging resistance of maize. Plant Growth Regul. 2017;84(2):317–32.

[143] Si T, Wang X, Huang M, Cai J, Zhou Q, Dai T, Jiang D. Double benefits of mechanical wounding in enhancing chilling tolerance and lodging resistance in wheat plants. Plant Biol. 2019;21(5):813–24. 30977948 10.1111/plb.12995

[144] Latimer JG. Mechanical conditioning to control plant size. HortTechnology. 1998;8(4):529–34.

[145] Graham T, Wheeler R. Mechanical stimulation controls canopy architecture and improves volume utilization efficiency in bioregenerative life-support candidate crops. Open Agric. 2017;2(Iss 1):42–51. 10.1515/opag-2017-0004 PMC6839710 31709306

[146] Iida H. Mugifumi, a beneficial farm work of adding mechanical stress by treading to wheat and barley seedlings. Front Plant Sci. 2014;5: 453. 25309553 10.3389/fpls.2014.00453 PMC4162469

[147] Feng L, Wang R, Wang R, Xu Q, Yang Y. Life cycle assessment of rice-duck co-culture systems. Ecosystem Health and Sustainability. 2024;10:10.

[148] Zheng H, Huang H, Chen C, Fu Z, Xu H, Tan S, She W, Liao X, Tang J. Traditional symbiotic farming technology in China promotes the sustainability of a flooded rice production system. Sustain Sci. 2016;12(1):155–61.

[149] Huang Z-X, Zhang J-E, Liang K-M, Quan G-M, Zhao B-L. Mechanical stimulation of duck on rice phyto-morphology in rice-duck farming system. Chin J Eco-Agric. 2012;20(6):717–22.

[150] Yang C, Han N, Liu M, Wei C, Mao R, Chen C. Effects of long-term different-scale rice-duck farming on the growth and yield of paddy rice. J Sci Food Agric. 2024;104(6):3729–35. 38160259 10.1002/jsfa.13257

[151] Markovic D, Colzi I, Taiti C, Ray S, Scalone R, Gregory Ali J, Mancuso S, Ninkovic V. Airborne signals synchronize the defenses of neighboring plants in response to touch. J Exp Bot. 2019;70(2):691–700. 30380091 10.1093/jxb/ery375 PMC6322579

[152] Delone NL, Berkovich YA, Smolyanina SO, Zimina NV, Davydova NV, Solovyev AA, Bolshakova LS. Vibration-induced stimulation of wheat growth. Dokl Biol Sci. 2010;434:332–4. 20963657 10.1134/S001249661005011X

[153] Crook MJ, Ennos AR. Mechanical differences between free-standing and supported wheat plants. Triticum aestivum L Annals of Botany. 1996;77:197–202.

[154] Dignac M-F, Derrien D, Barré P, Barot S, Cécillon L, Chenu C, Chevallier T, Freschet GT, Garnier P, Guenet B, Hedde M. Increasing soil carbon storage: mechanisms, effects of agricultural practices and proxies. A review Agronomy for Sustainable Development. 2017;37:14.

[155] Dijkstra FA, Zhu B, Cheng W. Root effects on soil organic carbon: a double-edged sword. New Phytol. 2021;230(1):60–5. 33197279 10.1111/nph.17082

[156] Pandey BK, Bennett MJ. Uncovering root compaction response mechanisms: new insights and opportunities. J Exp Bot. 2024;75(2):578–83. 37950742 10.1093/jxb/erad389 PMC10773992

[157] Hedden P. The genes of the Green Revolution. Trends Genet. 2003;19(1):5–9. 12493241 10.1016/s0168-9525(02)00009-4

[158] Khush GS. Green revolution: preparing for the 21st century. Genome. 1999;42:646–55. 10464789

[159] Jatayev S, Sukhikh I, Vavilova V, Smolenskaya SE, Goncharov NP, Kurishbayev A, Zotova L, Absattarova A, Serikbay D, Hu YG, Borisjuk N. Green revolution “stumbles” in a dry environment: Dwarf wheat with Rht genes fails to produce higher grain yield than taller plants under drought. Plant Cell Environ. 2020;43(10):2355–64. 32515827 10.1111/pce.13819

[160] Vikram P, Swamy BP, Dixit S, Singh R, Singh BP, Miro B, Kohli A, Henry A, Singh NK, Kumar A. Drought susceptibility of modern rice varieties: an effect of linkage of drought tolerance with undesirable traits. Sci Rep. 2015;5:14799. 26458744 10.1038/srep14799 PMC4602206

[161] Sun Q, Liu X, Yang J, Liu W, Du Q, Wang H, Fu C, Li WX. MicroRNA528 affects lodging resistance of maize by regulating lignin biosynthesis under nitrogen-luxury conditions. Mol Plant. 2018;11(6):806–14. 29597009 10.1016/j.molp.2018.03.013

[162] Le Gall H, Philippe F, Domon JM, Gillet F, Pelloux J, Rayon C. Cell wall metabolism in response to abiotic stress. Plants. 2015;4(1):112–66. 27135320 10.3390/plants4010112 PMC4844334

[163] Lionetti V, Cervone F, Bellincampi D. Methyl esterification of pectin plays a role during plant-pathogen interactions and affects plant resistance to diseases. J Plant Physiol. 2012;169(16):1623–30. 22717136 10.1016/j.jplph.2012.05.006

[164] Wu HC, Bulgakov VP, Jinn TL. Pectin methylesterases: Cell wall remodeling proteins are required for plant response to heat stress. Front Plant Sci. 2018;9: 1612. 30459794 10.3389/fpls.2018.01612 PMC6232315

[165] Ghosh R, Barbacci A, Leblanc-Fournier N. Mechanostimulation: a promising alternative for sustainable agriculture practices. J Exp Bot. 2021;72(8):2877–88. 33512423 10.1093/jxb/erab036

[166] Hilker M, Schwachtje J, Baier M, Balazadeh S, Baurle I, Geiselhardt S, Hincha DK, Kunze R, Mueller-Roeber B, Rillig MC, Rolff J. Priming and memory of stress responses in organisms lacking a nervous system. Biol Rev. 2016;91(4):1118–33. 26289992 10.1111/brv.12215

[167] Mauch-Mani B, Baccelli I, Luna E, Flors V. Defense priming: An adaptive part of induced resistance. Annu Rev Plant Biol. 2017;68:485–512. 28226238 10.1146/annurev-arplant-042916-041132

